# Taxonomy and Phylogeny of *Lepiota* Sect. *Stenosporae* (Verrucosporaceae) from Northeast China, with Six New Species and One New Record

**DOI:** 10.3390/jof12050355

**Published:** 2026-05-12

**Authors:** Xian-Yan Zhou, Tolgor Bau

**Affiliations:** Key Laboratory of Edible Fungal Resources and Utilization (North), Ministry of Agriculture and Rural Affairs, Jilin Agricultural University, Changchun 130118, China

**Keywords:** Agaricaceae, cryptic diversity, integrative taxonomy, species delimitation

## Abstract

*Lepiota* sect. *Stenosporae* is characterized by a trichodermal or cutis-like pileus covering and spurred basidiospores. Although macroscopic similarities among its members complicate field identification, species can be delimited by combining multi-locus (ITS, nrLSU, *rpb2*, and *tef1-α*) phylogenetic analyses with distinct micro-morphological features. Using this integrative approach, we investigated specimens of *sect. Stenosporae* collected from Northeast China. A total of 12 species were successfully delimited, including six species new to science (*Lepiota dolichospora*, *L. hongshiensis*, *L. jilinensis*, *L. microstenospora*, *L. sinocastanea*, and *L. sirupa*) and one new record for China (*L. grangei*). Comprehensive morphological descriptions and line-drawing illustrations of microscopic features are provided for all recognized taxa. These findings expand the known species diversity of *Lepiota* in China and contribute morphological and molecular data for further systematic studies of this fungal group.

## 1. Introduction

The genus *Lepiota* (Pers.) Gray represents one of the most species-rich lineages within the family *Verrucosporaceae* [1]. It typically encompasses saprotrophic fungi that produce small to medium-sized, fragile basidiomata with a white spore print. These species are widely distributed across global temperate and tropical regions, commonly inhabiting forest humus layers and soil [2]. Due to their high diversity in macromorphology, coloration, and microscopic features—as well as the presence of amatoxins in certain taxa [3]—*Lepiota* has long been a focal point and a significant challenge in fungal taxonomy [4,5,6,7,8,9,10,11].

At the infrageneric level, the traditional sectional classification of the genus *Lepiota* has primarily relied on the microstructure of the pileus covering and the morphological characteristics of basidiospores [12,13,14,15]. Recent taxonomic re-assessments combining multi-locus phylogeny and morphological data [10] have resolved the long-standing polyphyly within the genus, clearly defining seven independent monophyletic clades. Based on the pileus covering type and basidiospore shape, these sections are distinguished as follows: sect. *Cristatae* Kühner ex Wasser features a hymeniform pileus covering and ellipsoid or spurred basidiospores; sect. *Eriophorae* (Bon) Reschke & Sarawi is characterized by a pileus covering composed of oblong to spherical cells and small ellipsoid basidiospores; sect. *Fuscovinaceae* Bon & Candusso currently accommodates only *L. fuscovinacea* F.H. Møller & J.E. Lange, a species unique within the genus for its fibrillose basidiomata and the absence of clamp connections; sect. *Helveolae* (Bon & Boiffard) Bon has a trichodermal pileus covering lacking a hymeniform basal layer, with ellipsoid to ovoid basidiospores; sect. *Lepiota* (Pers.) Gray possesses a trichodermal pileus covering with a hymeniform basal layer and ellipsoid, amygdaloid, or fusiform basidiospores; sect. *Lilaceae* Bon has a hymeniform pileus covering and ellipsoid basidiospores and sect. *Stenosporae* J.E. Lange ex Reschke & Sarawi is distinguished by a trichodermal or cutis-like pileus covering and characteristically spurred basidiospores.

Within this framework, *Lepiota* sect. *Stenosporae* stands out due to its distinctive spurred basidiospores and trichodermal or cutis-like pileus covering. However, the typification of this section has recently undergone a significant revision. Historically, *L. pseudofelina* J.E. Lange was designated as the type species [16]. Nevertheless, nomenclatural tracing indicates that this species was not included in the original concept of the section [17], and multigene phylogenetic analyses have revealed that *L. pseudofelina* actually belongs to *Lepiota* sect. *Lepiota*. To resolve this discrepancy and maintain taxonomic stability, a recent comprehensive phylogenomic re-assessment [10] designated *L. castanea* Quél. as the new type species for sect. *Stenosporae*. Unlike *L. pseudofelina*, *L. castanea* forms a highly stable, strongly supported monophyletic core in phylogenetic trees. Furthermore, this species perfectly aligns with the original taxonomic scope and the anatomical definition of the section due to its typical spurred basidiospores and classic trichoderm pileipellis [10]. Consequently, our study follows this updated taxonomic framework.

Although the taxonomic and nomenclatural framework of sect. *Stenosporae* has been recently stabilized; species within this group exhibit pronounced similarities in their macroscopic features, rendering field identification highly challenging. In China, previous taxonomic studies have recorded eight species in this section: *L. castanea*, *L. erythrosticta* (Berk. & Broome) Sacc., *L. ignicolor* Bres., *L. luteocastanea* E. Horak, *L. mandarina* Jun F. Liang & Zhu L. Yang, *L. subcastanea* Jun F. Liang & Zhu L. Yang, *L. subcitrophylla* Hongo, and *L. tomentella* J.E. Lange [18,19]. Currently, *L. erythrosticta* and *L. luteocastanea* still lack reliable molecular data support. Specifically in Northeast China, the recent literature has documented only three species (*L. mandarina*, *L. castanea*, and *L. subcastanea*) [19]. These contributions provide valuable baseline data for evaluating the fungal resources of the region. Building upon this foundation, the application of modern multi-locus phylogenetic approaches is essential to more comprehensively reveal the actual species composition and diversity in this area.

To address this gap, the present study details the results of an intensive four-year field investigation conducted in Northeast China. By employing an integrative taxonomic approach that combines thorough morphological observations with multi-locus phylogenetic analyses, 12 species of sect. *Stenosporae* were successfully delimited from this region. Among them, six are described as species new to science, one is reported as a new record for China, and the remaining five are identified as known species (including *L. tomentella* J.E. Lange and *L. pilodes* Vellinga & Huijser, newly discovered in this region). Comprehensive morphological descriptions and line-drawing illustrations of microscopic features are provided for all recognized taxa.

## 2. Materials and Methods

### 2.1. Morphological Studies

Specimens were collected from Jilin Province and Heilongjiang Province, Northeast China, from July 2023 to August 2025. Macro-morphological characters and ecological data were recorded in situ, supplemented by high-resolution photographs of the basidiomata in their natural habitats. Color changes upon bruising or injury were documented; all color descriptions were based on a standard color chart [20]. Morphological descriptions follow established taxonomic standards [12,21]. The dried specimens are deposited in the Fungarium of Jilin Agricultural University (HMJAU/FJAU).

Global distribution data for specific taxa were retrieved and verified using the Global Biodiversity Information Facility (GBIF; https://www.gbif.org, accessed on 8 May 2026) [22].

### 2.2. DNA Extraction, PCR Amplification, Sequencing

Total genomic DNA was extracted from fresh or dried specimens using the NuClean PlantGen DNA Kit (CWBIO, Beijing, China). Four gene fragments were amplified via PCR: the internal transcribed spacer (ITS), the nuclear ribosomal large subunit (nrLSU), the second largest subunit of RNA polymerase II (*rpb2*), and the translation elongation factor 1-alpha (*tef1-*α). The primer pairs used were ITS1F/ITS4 [23,24], LR0R/LR5 [25], 6F/RPB2-7.1R [26], and EF1-983F/EF1-1567R [27], respectively. PCR protocols and thermal cycling conditions were based on previous studies [28]. Amplicons were sequenced by Sangon Biotech (Shanghai, China), and all resulting sequences were deposited in GenBank.

### 2.3. Phylogenetic Studies

To evaluate the phylogenetic relationships and accurately delimit species within *Lepiota* sect. *Stenosporae*, a combined multi-locus dataset (ITS, nrLSU, *rpb2*, and *tef1-α*) was assembled. The dataset comprised a total of 173 ITS, 60 nrLSU, 36 *rpb2*, and 24 *tef1-α* sequences (accession numbers are provided in Table 1). Among these, 150 sequences were newly generated in this study (88 ITS, 29 nrLSU, 13 *rpb2*, and 20 *tef1-α*), while the remaining sequences were retrieved from the GenBank database. Based on established taxonomic frameworks for *Lepiota* [10], representative species from other sections of *Lepiota* and the closely related genus *Echinoderma* were included to construct the phylogenetic tree of *Lepiota* sect. *Stenosporae*. Sequences of *Cystolepiota* s.l. were selected as the outgroup to root the phylogenetic trees.

**Table 1:** Information on DNA sequences used in the phylogenetic analyses. Sequences newly generated in this study are shown in bold. “—” means data not available. Capital letters after voucher represent Holotype “H”.

Taxon	Voucher	Origin	ITS	LSU	*rpb2*	*tef1*	References
*Cystolepiota hongshiensis*	HMJAU68205	China	OR947187	OR960533	PP465917	—	[28]
*C. luteosquamulosa*	HMJAU67711	China	OR233619	OR240263	PP465910	—	[28]
*Echinoderma “eriophorum”*	RITF2533	China	MK685378	MK685376	MK705800	—	Direct Submission
*E. asperum*	HAW: JKS142	USA	MK412598	—	—	—	[29]
***E. asperum***	**HMJAU37543**	**China**	**PZ184987**	**—**	**—**	**—**	**This study**
*E. flavidoasperum*	KUN-HKAS 87905	China	MN810147	MN810098	MN820969	MN820903	[30]
*E. hystrix*	C-F-13684 H	Denmark	PQ152708	—	—	—	[11]
*E. perplexum*	KaiR1713	Germany	PP594623	PP594751	PP841165	—	[10]
***Lepiota*** **aff.** ***castanea***	**FJAU78168**	**China**	**PZ185037**	**PZ185090**	**—**	**PZ213593**	**This study**
***L.*** **aff.** ***castanea***	**FJAU78195**	**China**	**PZ185027**	**PZ185087**	**—**	**—**	**This study**
***L.*** **aff.** ***castanea***	**FJAU78167**	**China**	**PZ185040**	**PZ185092**	**—**	**PZ213594**	**This study**
***L.*** **aff.** ***castanea***	**FJAU78281**	**China**	**PZ185019**	**—**	**—**	**—**	**This study**
***L.*** **aff.** ***subcastanea***	**FJAU78179**	**China**	**PZ185000**	**PZ185077**	**—**	**PZ213592**	**This study**
***L.*** **aff.** ***subcastanea***	**FJAU78189**	**China**	**PZ185070**	**PZ185103**	**—**	**PZ213591**	**This study**
***L.*** **aff.** ***subcastanea***	**FJAU78286**	**China**	**PZ184994**	**—**	**—**	**—**	**This study**
***L.*** **aff.** ***subcastanea***	**FJAU78178**	**China**	**PZ185007**	**—**	**—**	**—**	**This study**
***L.*** **aff.** ***subcastanea***	**FJAU78193**	**China**	**PZ184992**	**—**	**—**	**—**	**This study**
***L.*** **aff.** ***subcastanea***	**FJAU78285**	**China**	**PZ185016**	**—**	**—**	**—**	**This study**
***L.*** **aff.** ***subcastanea***	**FJAU78177**	**China**	**PZ185020**	**—**	**—**	**—**	**This study**
***L.*** **aff.** ***subcastanea***	**FJAU78180**	**China**	**PZ185002**	**PZ185079**	**—**	**—**	**This study**
*L. alopochroa*	MFLU 09-0178	Thailand	HQ647294	—	—	—	[7]
*L. andegavensis*	8-X-1994, P.D.H. Roux 2121	France	AY176461	—	—	—	[4]
*L. andegavensis*	T1-2	Lebanon	MZ088077	—	—	—	Direct Submission
*L. atrobrunneodisca*	HSA 115 H	China	OP724226	OP724232	—	—	[31]
*L. aurantiicolor*	SeSa176 H	Benin	PP594563	PP594681	PP841188	—	[11]
*L. baiyunensis*	B22052705 H	China	OQ547186	OQ547188	—	—	[32]
***L. baiyunensis***	**FJAU78273**	**China**	**PZ185049**	**—**	**—**	**—**	**This study**
***L. baiyunensis***	**FJAU79130**	**China**	**PZ185072**	**—**	**—**	**—**	**This study**
***L. boudieri***	**FJAU78282**	**China**	**PZ185062**	**—**	**—**	**—**	**This study**
***L. boudieri***	**FJAU78221**	**China**	**PZ185017**	**—**	**—**	**—**	**This study**
*L. boudieri*	HKAS 5803	China	EU416280	—	—	—	[33]
*L. brunneoaurantia*	UEH-F0006 H	Pakistan	OR464184	—	—	—	[34]
***L. brunneoincarnata***	**FJAU78269**	**China**	**PZ185051**	**—**	**—**	**—**	**This study**
***L. brunneoincarnata***	**FJAU78256**	**China**	**PZ185014**	**—**	**—**	**—**	**This study**
*L. brunneoincarnata*	HMAS 63488	China	EU416302	—	—	—	[33]
*L. brunneoolivacea*	SeSa214 H	Benin	PP594574	PP594693	PP841201	—	[11]
*L. brunneopileata*	LAH:37842	Pakistan	OQ970543	—	—	—	[35]
*L. castanea*	N.J. Dam 97020	Netherlands	AY176463	—	—	—	[4]
***L. castanea***	**FJAU78164**	**China**	**PZ185052**	**PZ185095**	**PZ213573**	**PZ213585**	**This study**
***L. castanea***	**FJAU78163**	**China**	**PZ185038**	**PZ185091**	**—**	**—**	**This study**
***L. castanea***	**FJAU78165**	**China**	**PZ185004**	**—**	**—**	**—**	**This study**
*L. castanea*	HKAS 48817	China	EU416282	—	—	—	[33]
*L. castanea*	S7	Germany	OL527673	—	—	—	[3]
***L. castanea***	**FJAU78176**	**China**	**PZ185025**	**—**	**—**	**—**	**This study**
***L. castanea***	**FJAU78175**	**China**	**PZ185024**	**—**	**—**	**—**	**This study**
***L. castanea***	**FJAU78166**	**China**	**PZ184997**	**—**	**—**	**—**	**This study**
*L. castaneidisca*	E.C. Vellinga 2516 (UC)	USA	AF391065	—	—	—	[36]
*L.* cf. *erythrostricta*	CL_MART_06.091	Martinique	PP594625	—	—	—	[11]
*L. citrophylla*	HNL502981	Laos	KX711968	—	—	—	[9]
***L. citrophylla***	**FJAU78220**	**China**	**PZ185028**	**—**	**—**	**—**	**This study**
*L. cristata*	HKAS49258	China	EU081937	—	—	—	[37]
***L. cristata***	**FJAU79132**	**China**	**PZ185056**	**—**	**—**	**—**	**This study**
***L. cristata***	**FJAU79133**	**China**	**PZ185059**	**—**	**—**	**—**	**This study**
***L. dolichospora***	**FJAU78183 H**	**China**	**PZ185053**	**PZ185096**	**PZ213574**	**PZ213587**	**This study**
***L. dolichospora***	**FJAU78184**	**China**	**PZ185060**	**PZ185098**	**—**	**—**	**This study**
***L. dolichospora***	**FJAU78185**	**China**	**PZ185012**	**PZ185082**	**—**	**—**	**This study**
***L. dolichospora***	**FJAU78192**	**China**	**PZ185046**	**—**	**—**	**—**	**This study**
*L. elseae*	AH:40487 H	Spain	NR_158471	—	—	—	[38]
*L. faiaebravae*	BCN-IC11111501	Portugal	PP622390	—	—	—	[39]
*L. flavonigrescens*	SeSa305 H	Benin	PP594596	PP594717	PP841226	—	[11]
*L. flavostipitata*	SeSa182	Benin	PP594568	PP594686	PP841193	—	[11]
*L. fuscovinacea*	E.C. Vellinga 2255	Netherlands	AY176372	AY176373	—	—	[5]
*L. grangei*	KaiR1768	Germany	PP594624	PP594638	PP841166	—	[11]
***L. grangei***	**FJAU78231**	**China**	**PZ185018**	**—**	**—**	**—**	**This study**
***L. grangei***	**FJAU78230**	**China**	**PZ185071**	**PZ185104**	**—**	**—**	**This study**
*L. griseovirens*	SeSa91	Germany	PP594548	PP594665	PP841272	—	[11]
*L. helveola*	S.D. Russell HRL2181	Canada	MH979466	—	—	—	Direct Submission
***L. hongshiensis***	**FJAU78254 H**	**China**	**PZ185036**	**PZ185089**	**—**	**—**	**This study**
***L. hongshiensis***	**FJAU78255**	**China**	**PZ185023**	**PZ185086**	**—**	**—**	**This study**
***L. hongshiensis***	**FJAU78295**	**China**	**PZ185009**	**—**	**—**	**—**	**This study**
*L. ignicolor*	17X1999 H. A. Huijser	Netherlands	AY176472	—	—	—	[4]
***L. jilinensis***	**FJAU78248 H**	**China**	**PZ185021**	**PZ185085**	**PZ213570**	**PZ213580**	**This study**
***L. jilinensis***	**FJAU78249**	**China**	**PZ185035**	**PZ185088**	**PZ213571**	**PZ213579**	**This study**
*L. lahorensis*	T18/LAH:10002012 H	Pakistan	KT182475	—	—	—	[40]
*L. lilacea*	E.C. Vellinga 1873	UK	GQ203820	—	—	—	[41]
*L. lilaceostriata*	SeSa280 H	Benin	PP594588	PP594709	PP841217	—	[11]
*L. longisterigmata*	SeSa179 H	Benin	PP594565	PP594683	PP841190	—	[11]
*L. maerimensis*	MFLU 12-2036	Thailand	MW251839	MW251847	—	—	[42]
***L. magnispora***	**HMJAU33688**	**China**	**PZ184986**	**—**	**—**	**—**	**This study**
*L. magnispora*	Z.L. Yang 2521	China	AF391006	—	—	—	[36]
*L. mandarina*	HKAS 50028 H	China	KM214811	KM214816	—	—	[18]
*L. metulispora*	HMGID25584	Unknown	MK651632	—	—	—	Direct Submission
***L. metulispora***	**FJAU79138**	**China**	**PZ184988**	**—**	**—**	**—**	**This study**
***L. metulispora***	**FJAU79137**	**China**	**PZ184989**	**—**	**—**	**—**	**This study**
***L. microstenospora***	**FJAU78246 H**	**China**	**PZ185011**	**PZ185081**	**PZ213567**	**PZ213581**	**This study**
***L. microstenospora***	**FJAU78247**	**China**	**PZ185043**	**PZ185094**	**PZ213572**	**PZ213582**	**This study**
*L. neophana*	RITF2402	Unknown	MK651599	—	—	—	Direct Submission
***L. neophana***	**FJAU79131**	**China**	**PZ185055**	**—**	**—**	**—**	**This study**
*L. neophana*	E.C. Vellinga 3955 (UC)	USA	GQ203811	—	—	—	[41]
*L. omninoflava*	KUN-HKAS 106734 HT	China	MN810157	MN810092	MN820951	MN820923	[30]
*L. omninoflava*	HMJAU68258	China	OR936203	—	—	—	[28]
***L. pallidiochracea***	**FJAU79135**	**China**	**PZ185050**	**—**	**—**	**—**	**This study**
***L. pallidiochracea***	**FJAU79136**	**China**	**PZ185067**	**—**	**—**	**—**	**This study**
*L. pallidiochracea*	HKAS:45579	China	NR_158462	—	—	—	[7]
*L. pilodes*	SeSa382	Germany	PP594615	—	PP841248	—	[11]
***L. pilodes***	**FJAU78232**	**China**	**PZ185001**	**PZ185078**	**—**	**PZ213588**	**This study**
***L. pilodes***	**FJAU78283**	**China**	**PZ185048**	**—**	**—**	**—**	**This study**
*L. pilodes*	E.C. Vellinga 3234 (UC)	USA	EF080865	—	—	—	[43,44]
*L. poliochloodes*	MFLU 081272	Thailand	HQ647296	—	—	—	[7]
*L. pseudovenenosa*	SeSa302 H	Benin	PP594593	PP594714	PP841223	—	[11]
*L. rhodophylla*	UC(USA-CA):1860004 H	USA	NR_119624	—	—	—	[45]
***L. sinocastanea***	**FJAU78172**	**China**	**PZ185065**	**PZ185101**	**PZ213577**	**PZ213583**	**This study**
***L. sinocastanea***	**FJAU78160**	**China**	**PZ185039**	**—**	**—**	**—**	**This study**
***L. sinocastanea***	**FJAU78171 H**	**China**	**PZ185063**	**PZ185100**	**—**	**PZ213584**	**This study**
***L. sinocastanea***	**FJAU78162**	**China**	**PZ185044**	**—**	**—**	**—**	**This study**
***L. sinocastanea***	**FJAU78170**	**China**	**PZ185041**	**PZ185093**	**—**	**PZ213595**	**This study**
***L. sinocastanea***	**FJAU78161**	**China**	**PZ184998**	**PZ185076**	**—**	**PZ213564**	**This study**
***L. sirupa***	**FJAU78262**	**China**	**PZ185057**	**—**	**—**	**—**	**This study**
***L. sirupa***	**FJAU78261 H**	**China**	**PZ185058**	**PZ185097**	**PZ213575**	**PZ213586**	**This study**
*L. sosuensis*	JBSD:CA3	Dominican Republic	NR_184875	—	—	—	[46]
*L. spiculata*	JBSD:127426	Dominican Republic	MK696156	MK696155	MK696576	MK696577	[47]
*L. squamulodiffracta*	CA21 HT	Dominican Republic	KR022006	—	—	—	[46]
*L. squamulosa*	HMJAU68251	China	OR936197	—	—	—	[28]
***L. squamulosa***	**FJAU79134**	**China**	**PZ185047**	**—**	**—**	**—**	**This study**
*L. subalba*	CWU(MYC)8416	Ukraine	OK041522	—	—	—	[48]
*L. subalba*	SeSa11	Austria	OL527683	—	—	—	[48]
*L. subcastanea*	HMJAU 3889	China	KM214814	—	—	—	[18]
*L. subcastanea*	HKAS 45633 H	China	KM214812	KM214817	—	—	[18]
*L. subcastanea*	HKAS49183	China	MK651652	MK685373	—	—	Direct Submission
***L. tomentella***	**FJAU78152**	**China**	**PZ185010**	**PZ185080**	**PZ213566**	**PZ213589**	**This study**
***L. tomentella***	**FJAU78159**	**China**	**PZ184999**	**—**	**—**	**—**	**This study**
***L. tomentella***	**FJAU78157**	**China**	**PZ185022**	**—**	**—**	**—**	**This study**
***L. tomentella***	**FJAU78158**	**China**	**PZ185045**	**—**	**—**	**—**	**This study**
***L. tomentella***	**FJAU78151**	**China**	**PZ185061**	**PZ185099**	**PZ213576**	**PZ213590**	**This study**
*L. tomentella*	RITF570	Unknown	MK651653	—	—	—	Direct Submission
***L. tomentella***	**FJAU78154**	**China**	**PZ185008**	**—**	**—**	**—**	**This study**
***L. tomentella***	**FJAU78155**	**China**	**PZ185005**	**—**	**—**	**—**	**This study**
***L. tomentella***	**FJAU78153**	**China**	**PZ185015**	**—**	**—**	**—**	**This study**
*L. tomentella*	SeSa58	Germany	PP594536	PP594652	PP841259	—	[11]
*L. tomentella*	H.A.Huijser (L)	Netherlands	EF080868	—	—	—	[43]
*L. tyrianthina*	SeSa213	Benin	PP594573	PP594692	PP841200	—	[11]
*L. vellingana*	MCR09	Pakistan	HE974764	—	—	—	[49]
*L. xanthophylla*	E.C. Vellinga 2240 (L)	Netherlands	AY176405	AY176406	—	—	[5]
*Lepiota* sp.	CUH AM839	India	OR594353	—	—	—	Direct Submission
*Lepiota* sp.	PS201885	Thailand	OP020473	—	—	—	Direct Submission
*Lepiota* sp.	110114MFBPC084	China	MW488365	—	—	—	Direct Submission
*Lepiota* sp.	MCVE 480	Unknown	FJ998390	—	—	—	Direct Submission
*Lepiota* sp.	SeSa110	Benin	PP594556	PP594674	PP841180	—	[11]
*Lepiota* sp.	SeSa255	Benin	PP594582	PP594703	PP841211	—	[11]
*Lepiota* sp.	SeSa346	Benin	PP594608	PP594731	PP841240	—	[11]
*Lepiota* sp.	ZRL20180888	China	PV607760	PV607733	PV614778	PV614794	[50]
*Lepiota* sp.	HKAS 82453	China	MN810130	MN810085	—	—	[30]
*Lepiota* sp.	BG0014	Pakistan	OM809699	—	—	—	Direct Submission
*Lepiota* sp.	BG0015	Pakistan	OM811292	—	—	—	Direct Submission
*Lepiota* sp.	MFLU 09-0183	Thailand	HQ647297	—	—	—	Direct Submission
*Lepiota* sp.	E.C. Vellinga 2603	USA	AY176481	—	—	—	[4]
*Lepiota* sp.	E.C. Vellinga 2601 (UC)	USA	AY176479	—	—	—	[4]
*Lepiota* sp.	S.D. Russell iNaturalist #63266348	USA	OM522742	—	—	—	Direct Submission
*Lepiota* sp.	S.D. Russell iNaturalist # 8607856	USA	MN906140	—	—	—	Direct Submission
*Lepiota* sp.	MycoMap #7207	USA	OM522727	—	—	—	Direct Submission
***Lepiota*** **sp.**	**FJAU78209**	**China**	**PZ185013**	**PZ185084**	**PZ213569**	**PZ213596**	**This study**
***Lepiota*** **sp.**	**FJAU78210**	**China**	**PZ185068**	**PZ185102**	**PZ213578**	**—**	**This study**
***Lepiota*** **sp.**	**FJAU78191**	**China**	**PZ185026**	**—**	**—**	**—**	**This study**
***Lepiota*** **sp.**	**FJAU78264**	**China**	**PZ185029**	**—**	**—**	**—**	**This study**
***Lepiota*** **sp.**	**FJAU78190**	**China**	**PZ184996**	**—**	**—**	**—**	**This study**
***Lepiota*** **sp.**	**FJAU78181**	**China**	**PZ185006**	**—**	**—**	**—**	**This study**
***Lepiota*** **sp.**	**FJAU78188**	**China**	**PZ184993**	**—**	**—**	**—**	**This study**
***Lepiota*** **sp.**	**FJAU78298**	**China**	**PZ185034**	**—**	**—**	**—**	**This study**
***Lepiota*** **sp.**	**FJAU78268**	**China**	**PZ185033**	**—**	**—**	**—**	**This study**
***Lepiota*** **sp.**	**FJAU78266**	**China**	**PZ185030**	**—**	**—**	**—**	**This study**
***Lepiota*** **sp.**	**FJAU25097**	**China**	**PZ184990**	**—**	**—**	**—**	**This study**
***Lepiota*** **sp.**	**FJAU78270**	**China**	**PZ185069**	**—**	**—**	**—**	**This study**
***Lepiota*** **sp.**	**FJAU78252**	**China**	**PZ184995**	**—**	**—**	**—**	**This study**
***Lepiota*** **sp.**	**FJAU78253**	**China**	**PZ185042**	**—**	**—**	**—**	**This study**
***Lepiota*** **sp.**	**FJAU78267**	**China**	**PZ185032**	**—**	**—**	**—**	**This study**
***Lepiota*** **sp.**	**FJAU78257**	**China**	**PZ185003**	**—**	**—**	**—**	**This study**
***Lepiota*** **sp.**	**FJAU78156**	**China**	**PZ185054**	**—**	**—**	**—**	**This study**
***Lepiota*** **sp.**	**FJAU78272**	**China**	**PZ185066**	**—**	**—**	**—**	**This study**
***Lepiota*** **sp.**	**FJAU78259**	**China**	**PZ185031**	**—**	**—**	**—**	**This study**
***Lepiota*** **sp.**	**HMJAU28816**	**China**	**PZ184991**	**—**	**—**	**—**	**This study**
***Lepiota*** **sp.**	**FJAU78229**	**China**	**PZ184985**	**PZ185083**	**PZ213568**	**PZ213565**	**This study**
***Melanophyllum eyrei***	**FJAU79139**	**China**	**PZ185064**	**—**	**—**	**—**	**This study**
*M. haematospermum*	K(M):176342	UK	MZ159454	—	—	—	Direct Submission

Sequences for each locus were aligned independently using MAFFT v.7.110 [51], followed by manual inspection and further optimization in MEGA v.7.0.26 [52]. Unreliable alignment regions and gaps were removed using the “-automated1” command in trimAl [53]. The aligned sequences were then concatenated using PhyloSuite v.2 [54], generating a combined alignment of 2780 base pairs (bp) in length. The sequence partitions were defined as follows: ITS (1–710 bp), nrLSU (711–1579 bp), *rpb2* (1580–2232 bp), and *tef1-α* (2233–2780 bp). Gaps were treated as missing data.

Phylogenetic reconstructions were performed using Maximum Likelihood (ML) and Bayesian Inference (BI) methods. To address the evolutionary heterogeneity among different gene regions, partitioned analyses were applied to the combined dataset. For the ML analysis, ModelFinder v.3.0.1 [55] was used to select the best-fit substitution models under the Akaike Information Criterion (AIC). The ML tree was inferred using IQ-TREE v.3.0.1 [56] with an edge-linked partition model. The selected best-fit models were TVM+F+I+R5 for ITS, GTR+F+I+R2 for nrLSU, TIM2+R3 for *rpb2*, and TIM3+I+R3 for *tef1-α*. Branch support was assessed using 1000 ultrafast bootstraps [57] and the Shimodaira–Hasegawa approximate likelihood-ratio test (SH-aLRT) [58].

For the BI analysis, ModelFinder v.3.0.1 [55] was employed to determine the optimal partition models based on the Bayesian Information Criterion (BIC), resulting in GTR+F+I+G4 for ITS and nrLSU, HKY+I+G4 for *rpb2*, and GTR+I+G4 for *tef1-α*. The BI analysis was conducted using MrBayes v.3.2.7a [59], running two parallel chains for 4,981,000 generations. The analysis was terminated when the average standard deviation of split frequencies (ASDSF) fell below 0.006. The initial 22% of the sampled data were discarded as burn-in. The resulting phylogenetic trees were visualized and annotated using FigTree v.1.4.3 [60] and tvBOT v.2.6.1 [61].

## 3. Results

### 3.1. Phylogenetic Analyses

**Figure 1:**
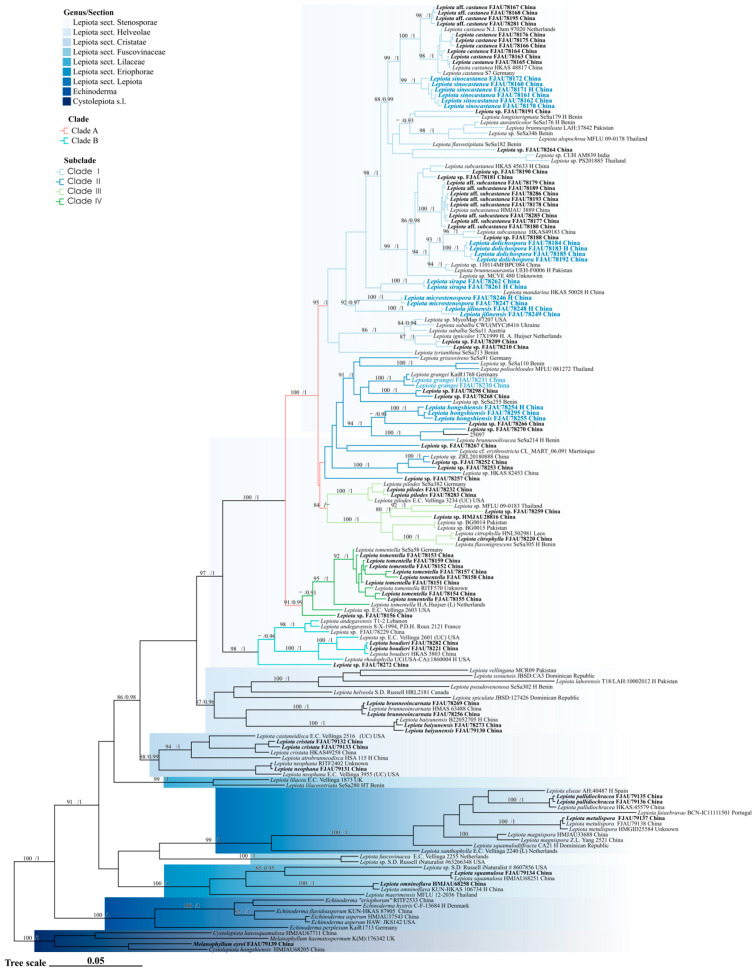
Bayesian phylogenetic tree of *Lepiota* sect. *Stenosporae* inferred from a combined dataset of ITS, nrLSU, *rpb2*, and *tef1-α* sequences. New species are indicated in bold blue, while new record for China are shown in blue. Sequences newly generated in this study are highlighted in bold black. Bayesian posterior probabilities (PP > 0.90) and Maximum Likelihood bootstrap support values (UFBoot > 80%) are indicated at the nodes (UFBoot/PP).

**Figure 2:**
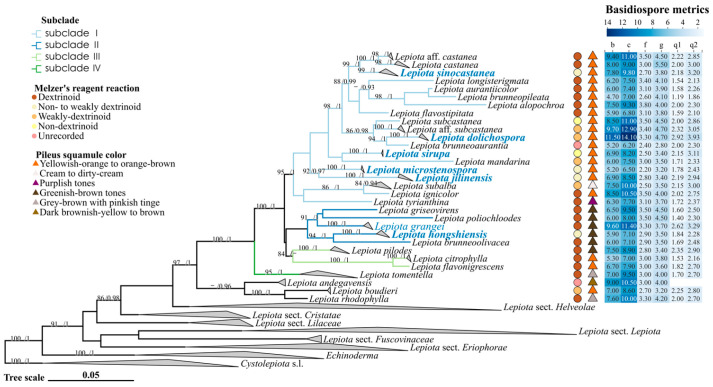
Bayesian phylogenetic tree of *Lepiota* sect. *Stenosporae* inferred from a combined dataset of ITS, nrLSU, *rpb2*, and *tef1-α* sequences. To emphasize the topological relationships within sect. *Stenosporae*, unidentified lineages (labeled as “*Lepiota* sp.”), and clades unrelated to this section have been collapsed. New species are indicated in bold blue, and new record for China are shown in blue. In the basidiospore metrics heatmap, b–c × f–g represents the range of basidiospore dimensions, while q1–q2 denotes the range of the Q value (length/width ratio). Bayesian posterior probabilities (PP > 0.90) and Maximum Likelihood bootstrap support values (UFBoot/80%) are indicated at the nodes (UFBoot/PP).

The phylogenetic relationships of *Lepiota* sect. *Stenosporae* were reconstructed using a concatenated dataset of ITS, nrLSU, *rpb2*, and *tef1-α*. The topologies generated by Maximum Likelihood (ML) and Bayesian Inference (BI) were congruent; thus, only the BI tree is presented (Figure 1 and Figure 2), with branch support values indicated at the nodes (UFBoot > 80%/PP > 0.90). In the phylogenetic reconstruction, newly described species are highlighted in bold blue, new records are indicated in blue, and newly generated sequences are marked in bold black. Using *Cystolepiota* s.l. as the outgroup, the *Lepiota* was resolved as a robust monophyletic group (91/1), within which sect. *Stenosporae* (97/1) was also recovered as a monophyletic lineage. Internally, the section diverged into two major well-supported clades, clade A (100/1) and clade B (98/1), which correlate with distinct types of pileus covering. Clade A is characterized by a trichodermal pileus covering, whereas clade B possesses a cutis-like pileus covering.

Within clade A (Figure 1), four distinct subclades (subclades I–IV) were identified. Subclade I (95/1) predominantly comprises species with orange-brown tones in their pileus scales, accommodating five new species described herein: *Lepiota sinocastanea*, *L. dolichospora*, *L. sirupa*, *L. microstenospora*, and *L. jilinensis*. Morphologically, the lamellae and stipe base of *L. sinocastanea* turn orange-red upon bruising, a characteristic rarely observed in other members of this subclade. *Lepiota dolichospora* is readily distinguished from its closely related species, *L. subcastanea* and *L. brunneoaurantia*, by its significantly larger and elongated basidiospores (Qav = 3.31). *Lepiota sirupa*, *L. microstenospora*, and *L. jilinensis* each occupy distinct phylogenetic lineages. Notably, *L. sirupa* possesses non-dextrinoid basidiospores, an uncommon trait within this subclade, and is currently known only from its type locality. *Lepiota microstenospora* and *L. jilinensis* are resolved as sister taxa; however, the former can be differentiated from the latter by its smaller basidiospores and narrower cheilocystidia. Subclades II and III are recovered as sister clades, although both their respective internal nodes and their most recent common ancestral node lack significant statistical support. Subclade II comprises species with greenish-brown pileus scales, including the new species *L. hongshiensis* and the new record for China, *L. grangei*. *Lepiota hongshiensis*, currently known only from its type locality, is phylogenetically most closely related to *L. brunneoolivacea* Sarawi. However, the lamellae of the latter shift to brownish-red upon maturation, a characteristic absent in *L. hongshiensis*. Although previously recorded from Europe, North America, South America, and various parts of Asia (including Western Asia, East Asia, and Southeast Asia), the occurrence of *L. grangei* in China is confirmed here for the first time based on molecular evidence. Subclade III (84/–) includes three species: *L. citrophylla* (Berk. & Broome) Sacc., *L. flavonigrescens* Sarawi & Reschke, and *L. pilodes*. Subclade IV (91/0.99) predominantly consists of *L. tomentella* along with two undetermined sequences labeled as *Lepiota* sp.

Conversely, clade B (98/1) (Figure 1) currently comprises only three known species, namely *L. andegavensis*, *L. boudieri*, and *L. rhodophylla*, along with three undetermined sequences labeled as *Lepiota* sp.

### 3.2. Taxonomy

***Lepiota dolichospora*** T. Bau & X.Y. Zhou, sp. nov.

MycoBank No: MB862979

**Figure 3:**
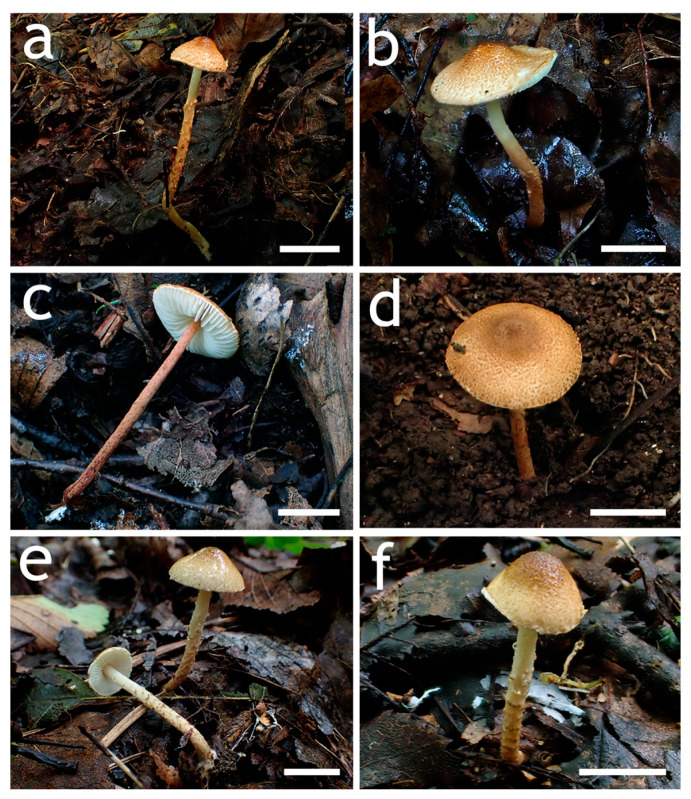
Basidiomata of *Lepiota* sect. *Stenosporae* species. (**a**–**c**) *L. dolichospora* ((**a**) FJAU78185; (**b**) FJAU78184; (**c**) FJAU78183); (**d**,**e**) *L. hongshiensis* ((**d**) FJAU78295: (**e**,**f**) FJAU78254). Scale bars = 1 cm.

**Figure 4:**
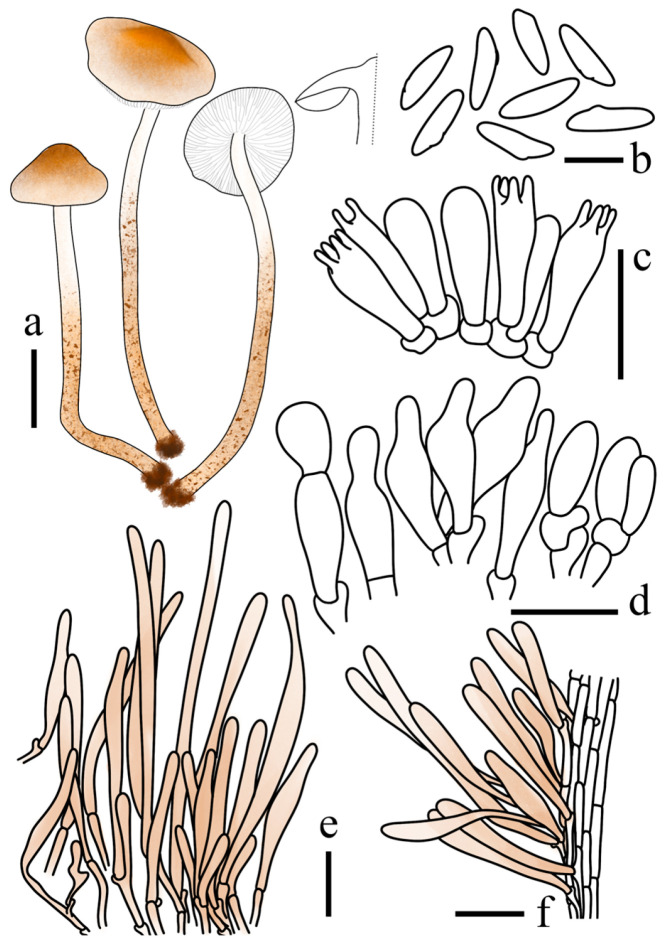
*Lepiota dolichospora* (FJAU78183, holotype). (**a**) Basidiomata; (**b**) Basidiospores; (**c**) Basidia; (**d**) Cheilocystidia; (**e**) Pileus covering; (**f**) stipe covering. Scale bars: (**a**) =1cm; (**b**) =10 µm; (**c**,**d**) =20 µm; (**e**,**f**) =50 µm.

Figure 3a–c and Figure 4

Holotype: CHINA. Jilin Province, Huadian City, Hongshi National Forest Park, 27 August 2023, X.Y. Zhou and T. Bau, FJAU78183.

Etymology: “*dolichospora*” is derived from the Greek ‘dolichos’ (long) and ‘spora’ (spore), referring to the remarkably elongated and slender basidiospores, which characterize this species within the section *Stenosporae*.

Diagnosis: *Lepiota dolichospora* is primarily distinguished by its small-sized basidiomata with a light orange to deep orange pileus covered in minute squamules; basidiospores that are remarkably elongated and spurred, measuring 11.5–14.1 × 3.3–4.7 μm with an exceptionally high average Q-value (Qav = 3.31) and showing a weakly dextrinoid reaction; cheilocystidia that are predominantly narrowly utriform to utriform; and a pileus covering structured as a trichoderm with slightly thick-walled terminal cells. It differs from *L. subcastanea* by its much longer and narrower spores (Qav = 2.48 in *L. subcastanea*) and from *L. brunneoaurantia* by its significantly larger spore dimensions.

Basidiomata small. Pileus 0.8–2.3 cm in diam., campanulate when young, expanding to plano-convex at maturity; surface white, densely covered in light orange (6A3–A5) to deep orange minute squamules; central squamules brownish orange (7C6–C8); margin with veil remnants concolorous with the squamules. Context thin, white, unchanging when bruised. Lamellae free,, crowded, interspersed with lamellulae of unequal length, ventricose, white to cream; edge entire, concolorous. Stipe 3.8–8.1 × 0.2–0.4 cm, subcylindrical, slightly thickening towards the base, white to orange white (6A2), with a distinct annular zone; surface nearly glabrous above the annular zone, lower portion densely ornamented with light orange (6A3–A5) to deep orange (6A6–A8) squamules arranged in interrupted annular bands. Odour and taste not recorded.

Basidiospores (40/2/2) 11.5–14.1 × 3.3–4.7 μm, avl × avw = 12.9 × 3.9 μm, Q = 2.92–3.93, Qav = 3.31, spurred, remarkably elongated, sub-triangular in side view, long-ellipsoid in frontal view; wall hyaline, thin, weakly dextrinoid. Basidia 17–27 × 6–11 μm, clavate, 4- (2-) spored, thin-walled. Lamella edge sterile. Cheilocystidia 21–38 × 5–11 μm, predominantly narrowly utriform to utriform, occasionally fusiform, hyaline, thin-walled. Pleurocystidia absent. Pileus covering a trichoderm; terminal cells 65–218 × 7–17 μm, erect, cylindrical to clavate, with rounded apices and slightly thick walls, containing light orange (6A3–A5) to greyish orange (6B3–B5) intracellular pigments. Stipe covering a cutis; terminal cells 32–180 × 9–16 μm, appressed, cylindrical to clavate, containing light orange (6A3–A5) to greyish orange (6B3–B5) intracellular pigments. Clamp connections present.

Habit and Habitat. Solitary on soil within the humus layer of broad-leaved forests dominated by *Betula* spp., *Fraxinus mandshurica* Rupr., and *Juglans mandshurica* Maxim.

Known distribution: Jilin Province, China.

Additional specimens examined: CHINA. Jilin Province, Jiaohe City, Shansongling, 24 July 2022, W.N. Hou, X. Wang, FJAU78185, FJAU78192; Huadian City, Hongshi National Forest Park, 16 August 2024, X.Y. Zhou FJAU78184.

Notes: *Lepiota dolichospora* is nested within the *L. subcastanea* complex of *Lepiota* sect. *Stenosporae*. This complex currently encompasses several taxa, including *L*. *subcastanea* Jun F. Liang & Zhu L. Yang [18], *L.* aff. *subcastanea*, *L*. *brunneoaurantia* Azeem & Jabeen [34], and *L*. *subcastanea* var. *bispora* Jun F. Liang & Zhu L. Yang [6].

Morphologically, *L. dolichospora* is best recognized by its exceptionally elongated, spurred basidiospores. It possesses the slenderest spores within the complex (Qav = 3.31), which readily distinguishes it from closely related taxa that exhibit relatively broader spore profiles, such as *L*. *subcastanea* (Qav = 2.48) [19], *L*. aff. *subcastanea* (Qav = 2.83), and *L*. *subcastanea* var. *bispora* (Qav = 2.35) [6].

Phylogenetically, *L. dolichospora* forms a well-supported, independent monophyletic lineage (100/1). Although it is recovered as a sister group to *L. brunneoaurantia*, the two are morphologically distinct; the latter possesses significantly smaller basidiospores (5.2–6.2 × 2.4–2.8 µm) compared to those of the new species (11.5–14.1 × 3.3–4.7 μm).

Within this complex, *L*. aff. *subcastanea* exhibits a distinct morphological transition. Despite being phylogenetically distinct from *L*. *dolichospora*, its spore length (averaging 11.0 × 3.9 μm) is intermediate between those of *L. subcastanea* and *L. dolichospora*. Furthermore, the most remarkable taxonomic trait of this lineage is the instability in sterigmata number, featuring a nearly equal proportion of 2-spored and 4-spored basidia—a condition exceptionally rare within sect. *Stenosporae*, where 4-spored basidia typically predominate.

This variation in basidia bears resemblance to *L*. *subcastanea* var. *bispora* [6] described from Tibet, which is characterized primarily by 2-spored basidia. However, the Tibetan variety possesses significantly broader basidiospores (up to 5.5 μm in width). Given that the variety remains poorly documented and lacks molecular data, it is currently difficult to determine whether *Lepiota* aff. *subcastanea* is conspecific with *L*. *subcastanea* var. *bispora*.

***Lepiota hongshiensis*** T. Bau & X.Y. Zhou, sp. nov.

MycoBank No: 862980

**Figure 5:**
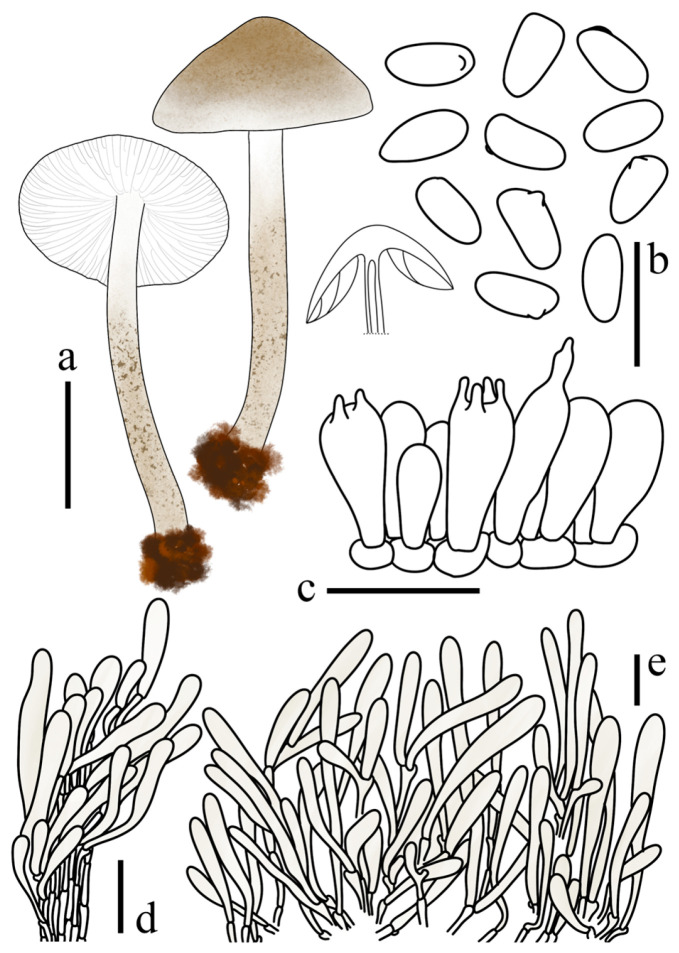
*Lepiota hongshiensis* (FJAU78254, holotype). (**a**) Basidiomata; (**b**) Basidiospores; (**c**) Basidia; (**d**) stipe covering; (**e**) Pileus covering. Scale bars: (**a**) =1 cm; (**b**) =10 µm; (**c**) =20 µm; (**d**,**e**) =50 µm.

Figure 3d–f and Figure 5

Holotype: CHINA. Jilin Province, Huadian City, Hongshi National Forest Park, 16 August 2024, Z. Q. Chen, FJAU78254.

Etymology: “*hongshiensis*” refers to the type locality, Hongshi National Forest Park in Huadian City, Jilin Province, China, where all known specimens of this species were collected.

Diagnosis: *Lepiota hongshiensis* is characterized by its small-sized basidiomata with a pileus densely covered in greyish yellow to olive brown minute squamules; a stipe surface ornamented with squamules arranged in interrupted annular bands; remarkably non-weakly dextrinoid and spurred basidiospores measuring 5.9–7.1 × 2.9–3.5 μm; notably absent or not distinctly differentiated cheilocystidia; and a trichodermal pileus covering.

Basidiomata small. Pileus 0.9–1.8 cm in diam., campanulate when young, then expanding to plano-convex; surface white, densely clothed in minute greyish yellow (4C4–C8) to olive brown (4D6–D8) squamules. Context thin, white, unchanging when bruised. Lamellae free, crowded, interspersed with lamellulae of unequal length, ventricose, white to cream; edge entire, concolorous. Stipe 2.9–3.4 × 0.2–0.3 cm, subcylindrical, slightly thickening towards the base, white to cream; surface nearly glabrous above the annular zone, lower portion densely ornamented with greyish yellow (4C4–C8) to olive brown (4D6–D8) squamules arranged in interrupted annular bands. Odour and taste not recorded.

Basidiospores (40/2/2) 5.9–7.1 × 2.9–3.5 μm, avl × avw = 6.6 × 3.2 μm, Q = 1.84–2.28, Qav = 2.01, spurred, sub-triangular in profile, base occasionally nearly straight, ellipsoid in frontal view; wall hyaline, smooth, non-to weakly dextrinoid. Basidia 15–27 × 5–10 μm, clavate, 4- (2-) spored, thin-walled. Lamella edge fertile. Cheilocystidia not distinctly differentiated. Pleurocystidia absent. Pileus covering a trichoderm; terminal cells 61–239 × 9–19 μm, erect, cylindrical to clavate, with rounded apices and slightly thick walls, containing greyish yellow (4B2–B3) to light blond (4C2–C4) intracellular pigments. Stipe covering a cutis; terminal cells 63–122 × 9–21 μm, appressed, cylindrical to clavate, containing light blond (4C2–C4) intracellular pigments. Clamp connections present.

Habit and Habitat. Solitary to scattered on soil within the humus layer of broad-leaved forests dominated by *Fraxinus mandshurica*, *Quercus mongolica* Fisch. ex Ledeb., *Juglans mandshurica*, *Betula* spp., and *Ulmus* spp.

Known distribution: Jilin Province, China.

Additional specimens examined: CHINA. Jilin Province, Huadian City, Hongshi National Forest Park, 23 August 2024, R.H. Lin and T. Bau, FJAU78255; 15 August 2025, Y.F. Han, FJAU78295.

Notes: Based on our microscopic examination of the two specimens of *Lepiota hongshiensis*, no differentiated terminal cells or protruding hymenial structures were observed along the lamellar edges. Consequently, this species is characterized by the absence of cheilocystidia.

Phylogenetically, *L*. *hongshiensis* is nested within a clade primarily composed of species with greenish-brown pileus tones, including *L*. *griseovirens* Maire, *L*. *poliochloodes* Vellinga & Huijser, *L. grangei* (Eyre) Kühner, *L*. *brunneoolivacea* Sarawi, and *L*. *pilodes* Vellinga & Huijser.

Morphologically, however, *L*. *hongshiensis* clearly differs from these close relatives by its persistently white to cream lamellae, unchanging context, and relatively small basidiospores (5.9–7.1 × 2.9–3.5 μm). In contrast, the initially white to cream lamellae of *L*. *griseovirens* develop orange-brown spots with age, and the species possesses significantly larger and broader spores [(6.0–)6.5–9.5(–11.0) × (3.0–)3.5–4.5 μm] [12]. Similarly, *L*. *poliochloodes* is differentiated by its context and lamellae, which turn pinkish to pale orange-brown [12]. *L*. *grangei* is characterized by dense, dark-brown punctate squamules on the lower stipe and markedly larger spores (9.6–11.4 × 3.3–3.7 μm). Furthermore, the lamellae of *L*. *brunneoolivacea* shift to brownish-red upon maturation [11], while *L*. *pilodes* develops orange-brown spots on the lamellae and forms larger spores [(7.0–)8.0–10.0(–11.5) × 3.0–4.0(–4.5) μm].

These morphological distinctions, particularly the stable color of the hymenophore and the specific spore dimensions, support the recognition of *L*. *hongshiensis* as an independent species within this lineage.

***Lepiota jilinensis*** T. Bau & X.Y. Zhou, sp. nov.

MycoBank No: 862981

**Figure 6:**
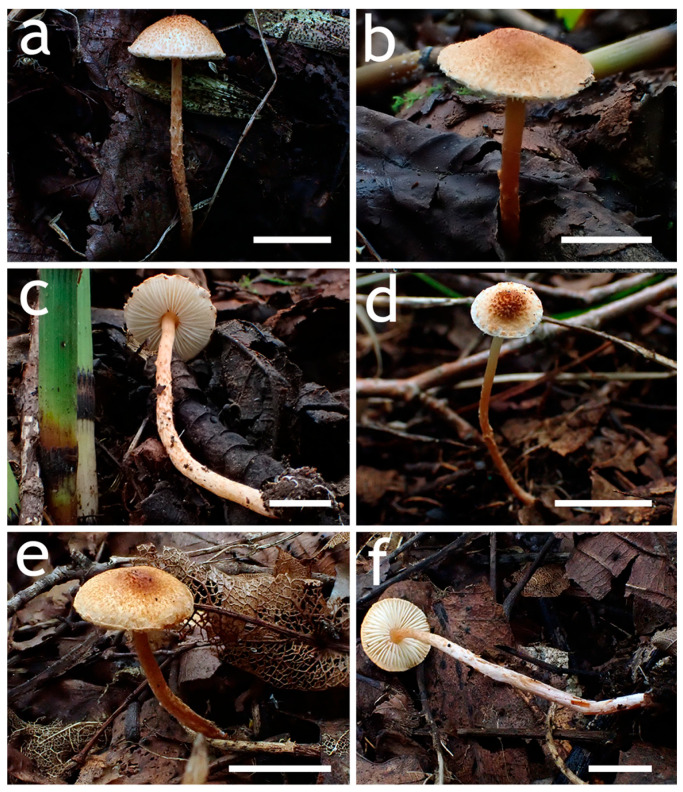
Basidiomata of *Lepiota* sect. *Stenosporae* species. (**a**–**c**) *L. jilinensis* ((**a**) FJAU78248; (**b**,**c**) FJAU78249); (**d**–**f**) *L. microstenospora* ((**d**) FJAU78246; (**e**,**f**) FJAU78247); (**a**–**f**) Scale bars = 1 cm.

**Figure 7:**
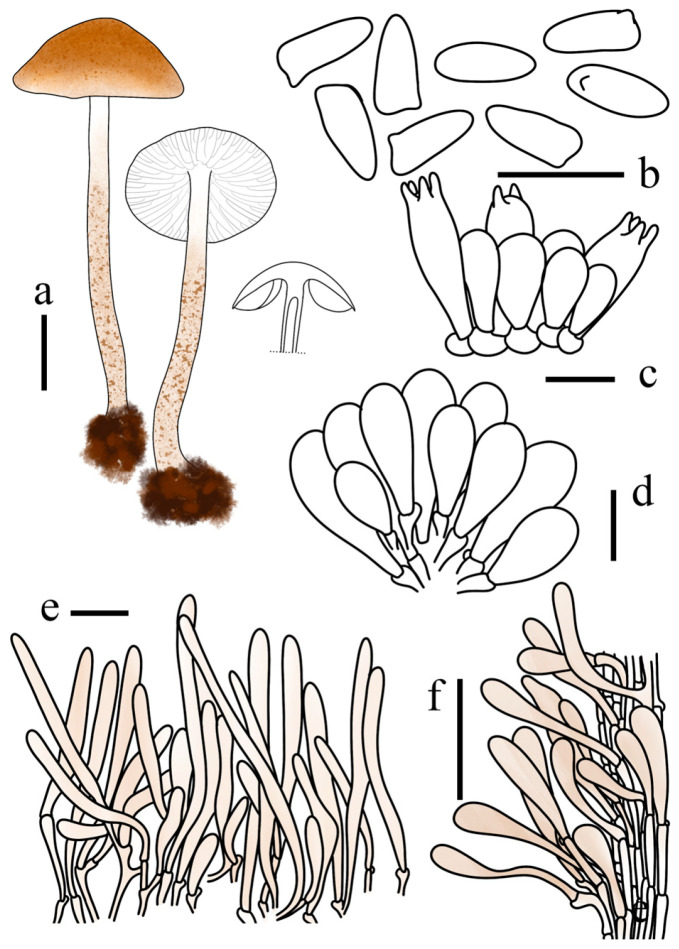
*Lepiota jilinensis* (FJAU78248, holotype). (**a**) Basidiomata; (**b**) Basidiospores; (**c**) Basidia; (**d**) Cheilocystidia; (**e**) Pileus covering; (**f**) stipe covering. Scale bars: (**a**) =1 cm; (**b**) =10 µm; (**c**,**d**) =20 µm; (**e**,**f**) =50 µm.

Figure 6a–c and Figure 7

Holotype: CHINA. Jilin Province, Jiaohe City, Qianjin Forest Farm, 25 August 2023, M. Liu and T. Bau, FJAU78248.

Etymology: “*jilinensis*” refers to Jilin Province, Northeast China, where all the specimens of this species were collected.

Diagnosis: *Lepiota jilinensis* is characterized by its pileus densely clothed in light orange to brownish orange squamules; a trichodermal pileus covering; spurred basidiospores that appear rectangular to sub-triangular in profile; and clavate to broadly clavate cheilocystidia.

Basidiomata small. Pileus 1–1.9 cm in diam., lenticular; surface white, adorned with light orange (6A3–A5) to brownish orange (7C6–C8) squamules; squamules densely aggregated and darker toward the disc. Context thin, white, unchanging when bruised. Lamellae free, crowded, alternating with several tiers of lamellulae, ventricose, white to cream; edge entire, concolorous. Stipe 4.1–5.0 × 0.1–0.2 cm, subcylindrical, light orange (6A3–A5) to brownish orange (7C6–C8); surface nearly glabrous above the annular zone, lower portion clothed in brownish orange (7C6–C8) squamules. Odour and taste not recorded.

Basidiospores (40/2/2) (6.6–) 6.9–8.5 × 2.8–3.4 μm, avl × avw = 7.6 × 3.1 μm, Q = 2.19–2.94, Qav = 2.64, spurred, rectangular to sub-triangular in profile, cylindrical to sub-fusiform in frontal view; supra-hilar area not depressed or occasionally slightly depressed; base more or less with an extended spur; ventral side sometimes weakly ventricose; dorsal side occasionally slightly depressed at the base; wall slightly thickened, hyaline, smooth, non- to weakly dextrinoid. Basidia 14–25 × 4–8 μm, clavate, 4- (2-) spored, thin-walled. Lamella edge sterile. Cheilocystidia 19–35 × 9–18 μm, clavate to broadly clavate, occasionally sphaeropedunculate, hyaline, thin-walled. Pleurocystidia absent. Pileus covering a trichoderm; terminal cells 70–306 × 9–19 μm, erect, cylindrical to clavate, with rounded apices and slightly thick walls, containing light orange (6A3–A5) to greyish orange (6B3–B5) intracellular pigments; with short clavate cells, containing greyish orange (6B3–B5) intracellular pigments. Stipe covering a cutis; terminal cells 50–167 × 7–15 μm, appressed, cylindrical to clavate, containing light orange (6A3–A5) to greyish orange (6B3–B5) intracellular pigments. Clamp connections present.

Habit and Habitat. Solitary to scattered on soil within the humus layer of broad-leaved forests dominated by *Fraxinus mandshurica*, *Quercus mongolica*, and *Juglans mandshurica*.

Known distribution: Jilin Province, China.

Additional specimens examined: CHINA. Jilin Province, Huadian City, Hongshi National Forest Park, 28 August 2023, Z.Q. Chen and T. Bau, FJAU78249.

Notes: *Lepiota jilinensis* is a characteristic orange-brown member of sect. *Stenosporae* that shares morphological similarity with several other lineages within the section. However, it can be reliably distinguished by a combination of basidiospore dimensions, Q values, and chemical reactions.

Within the orange-brown complex, *L*. *jilinensis* is primarily defined by its medium-sized, non- to weakly dextrinoid basidiospores. This feature readily separates it from the large-spored taxa, such as *L*. *dolichospora* and *L. subcastanea*, as well as from the relatively small-spored species like *L*. *microstenospora*.

Furthermore, although *L*. *jilinensis*, *L*. *sinocastanea*, and *L*. *sirupa* all possess medium-sized basidiospores, they are clearly distinct based on other characters. *L*. *sinocastanea* is distinguished by its lamellae discoloring upon bruising and *L*. *sirupa* by its cylindrical to narrowly clavate cheilocystidia. In contrast, the lamellae of *L*. *jilinensis* remain unchanged when injured, and it possesses clavate to broadly clavate cheilocystidia.

***Lepiota microstenospora*** T. Bau & X.Y. Zhou, sp. nov.

MycoBank No: 862982

**Figure 8:**
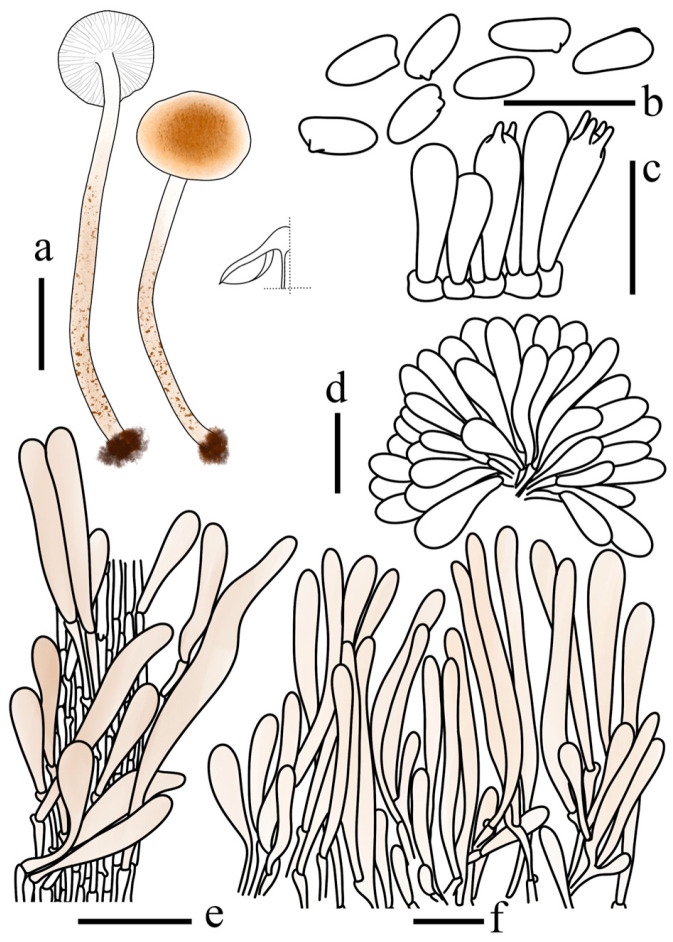
*Lepiota microstenospora* (FJAU78246, holotype). (**a**) Basidiomata; (**b**) Basidiospores; (**c**) Basidia; (**d**) Cheilocystidia; (**e**) stipe covering; (**f**) Pileus covering. Scale bars: (**a**) =1 cm; (**b**) =10 µm; (**c**,**d**) =20 µm; (**e**,**f**) =50 µm.

Figure 6d–f and Figure 8

Holotype: CHINA. Jilin Province, Jiaohe City, Qianjin Forest Farm, 23 July 2022, W.N. Hou, FJAU78246.

Etymology: “*microstenospora*” refers to the remarkably small basidiospores (5.7 × 2.8 μm), which represent the smallest spore dimensions recorded within the section *Stenosporae* to date.

Diagnosis: *Lepiota microstenospora* is primarily characterized by its pileus surface densely clothed in light orange to brownish orange, erect, fasciculate, and floccose squamules; a trichodermal pileus covering; remarkably small and spurred basidiospores (5.2–6.5 × 2.2–3.2 μm) that appear rectangular to sub-triangular in profile; and narrowly clavate to clavate cheilocystidia.

Basidiomata small. Pileus 0.7–1.2 cm in diam., plano-convex, with a distinct blunt umbo at the center; surface white, densely clothed in light orange (6A2–A5) to brownish orange (7C6–C8), erect, fasciculate, and floccose squamules. Context extremely thin, white, unchanging when bruised. Lamellae free, crowded, with lamellulae, ventricose, white to cream; edge entire, concolorous. Stipe 3.0–3.6 × 0.1–0.2 cm, subcylindrical, slightly thickening towards the base, white to light orange (6A2–A5) above the annular zone, light orange (6A2–A5) or brownish orange (7C6–C8) below; surface ornamented with concolorous floccose squamules. Stipe context reddish brown (9E5–E8). Odour and taste not recorded.

Basidiospores (40/2/2) 5.2–6.5 × 2.2–3.2 μm, avl × avw = 5.7 × 2.8 μm, Q = 1.78–2.43, Qav = 2.05, spurred, sub-triangular to rectangular in profile, oblong in frontal view; wall hyaline, smooth, non- to weakly dextrinoid. Basidia 18–22 × 4–8 μm, clavate, 4- (2-) spored, thin-walled. Lamella edge sterile. Cheilocystidia 23–38 × 5–9 μm, narrowly clavate to clavate, hyaline, thin-walled. Pleurocystidia absent. Pileus covering a trichoderm; terminal cells 89–247 × 6–15 μm, erect, cylindrical to clavate, with rounded apices and slightly thick walls, containing light orange (6A3–A5) to greyish orange (6B3–B5) intracellular pigments. Stipe covering a cutis; terminal cells 52–160 × 9–20 μm, appressed, cylindrical to clavate, containing light orange (6A3–A5) to greyish orange (6B3–B5) intracellular pigments. Clamp connections present.

Habit and Habitat. Solitary to scattered on soil within the humus layer of broad-leaved forests dominated by *Quercus mongolica*, *Juglans mandshurica*, *Betula* spp., and *Ulmus* spp.

Known distribution: Jilin Province, China.

Additional specimens examined: CHINA. Jilin Province, Jiaohe City, Qianjin Forest Farm, 25 August 2023, X. Wang and T. Bau, FJAU78247.

Notes: Although *Lepiota microstenospora* shares the orange-brown pileus coloration typical of other members in the *Lepiota* sect. *Stenosporae* complex, it is well separated by its exceptionally small basidiospores. Furthermore, the pileus center of this species features distinct, sub-erect, tufted, and dark tomentose squamules.

Within this complex, other small-spored species such as *L*. *brunneoaurantia*, *L*. *flavostipitata* Sarawi, and *L. brunneopileata* A. Rehman, Afshan, Usman & Khalid differ morphologically and chemically. *Lepiota brunneoaurantia* has markedly shorter and narrower cheilocystidia [(8.6–)8.9–13.1(–13.8) × (3.8–)4.2–5.9(–6.2) μm] [34]. In contrast to the non- to weakly dextrinoid basidiospores of *L*. *microstenospora*, those of *L*. *flavostipitata* and *L*. *brunneopileata* are distinctly dextrinoid [11,35]. Additionally, *L*. *brunneopileata* lacks the sub-erect, tufted squamules observed in the new species [35].

***Lepiota sinocastanea*** T. Bau & X.Y. Zhou, sp. nov.

MycoBank No: 862983

**Figure 9:**
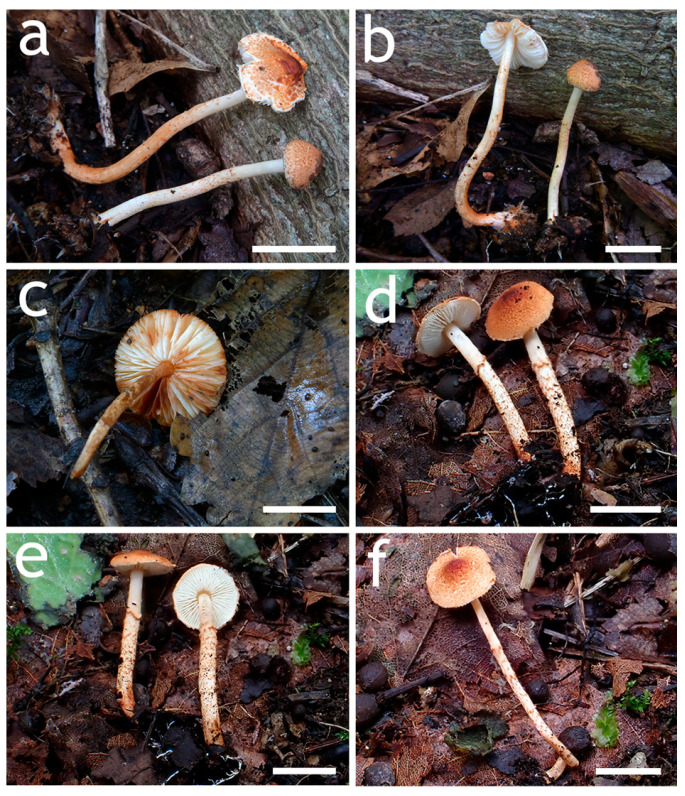
Basidiomata of *Lepiota* sect. *Stenosporae* species. (**a**–**c**) *L. sinocastanea*. ((**a**,**b**) FJAU78171; (**c**) FJAU78172); (**d**–**f**) *L. sirupa* ((**d**,**e**) FJAU78261; (**f**) FJAU78262). Scale bars = 1 cm.

**Figure 10:**
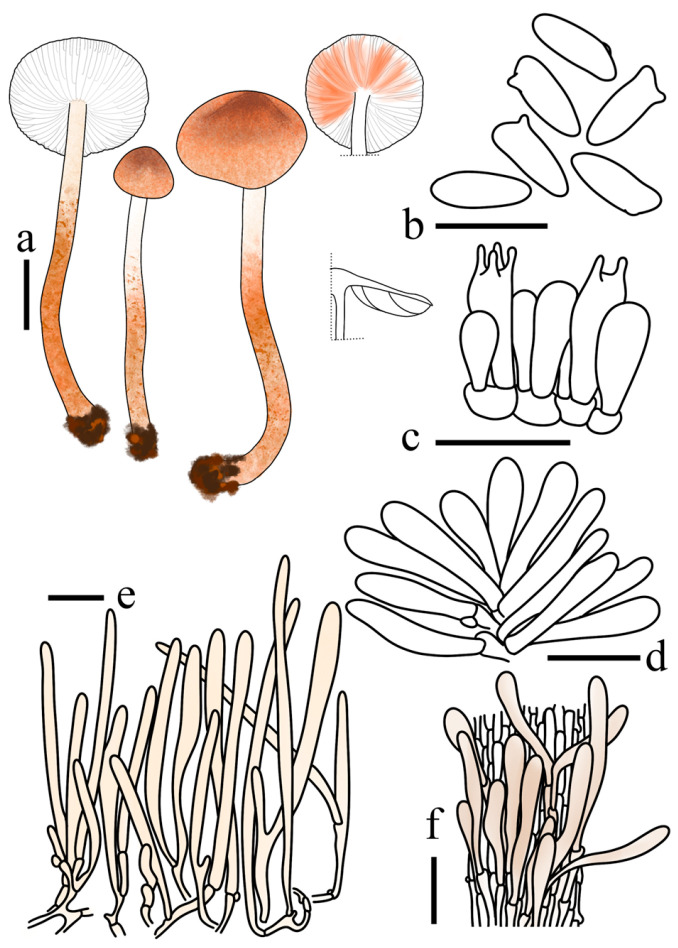
*Lepiota sinocastanea* (FJAU78171, holotype). (**a**) Basidiomata; (**b**) Basidiospores; (**c**) Basidia; (**d**) Cheilocystidia; (**e**) Pileus covering; (**f**) stipe covering. Scale bars: (**a**) =1 cm; (**b**) =10 µm; (**c**,**d**) =20 µm; (**e**,**f**) =50 µm.

Figure 9a–c and Figure 10

Holotype: CHINA. Jilin Province, Ji’an City, Wunufeng National Forest Park, 25 July 2025, X. Y. Zhou, FJAU78171.

Etymology: The epithet “*sinocastanea*” refers to the Chinese origin (Sino-) of the species and its morphological resemblance (-*castanea*) to the original description of *Lepiota castanea* Quélet.

Diagnosis: *Lepiota sinocastanea* is characterized by its small-sized basidiomata with light orange to brownish orange scales; lamellae white to cream, bruising light orange (6A2–A5) to greyish orange (6B3–B6) when damaged; spurred basidiospores measuring 7.8–9.8 × 2.7–3.8 μm, non- to weakly dextrinoid in Melzer’s reagent; and a distinct phylogenetic position at the base of the *L. castanea* complex. It differs from the European *L. castanea* s.l. by its relatively small spores and the non- to weaker dextrinoid reaction.

Basidiomata small. Pileus 0.6–1.7 cm in diam., obtuse conical when young, becoming plano-convex to applanate at maturity; surface white, with a dense pileus covering composed of light orange (6A3–A6) to brownish orange (7C6–C8) scales. Context thin, white. Lamellae free, crowded, interspersed with lamellulae, ventricose, white to cream, bruising light orange (6A2–A5) to greyish orange (6B3–B6) when damaged; edge entire, concolorous. Stipe 2.1–5.0 × 0.2–0.3 cm, subcylindrical, slightly thickening towards the base, white; surface nearly smooth above the annulus, lower portion densely covered with light orange (6A3–A6) to brownish orange (7C6–C8) fine granular scales arranged in discontinuous, zonate bands; base bruising greyish orange (6B3–B6) upon injury. Odour and taste not recorded.

Basidiospores (40/2/2) 7.8–9.8 × 2.7–3.8 μm, avl × avw = 8.6 × 3.3 μm, Q = 2.18–3.20, Qav = 2.59, sub-triangular in profile with a distinct spurred base, long-ellipsoid to sub-fusiform in frontal view; apex often slightly acute; wall hyaline, smooth, slightly thick, non- to weakly dextrinoid. Basidia 16–25 × 6–9 μm, 4-(2-)spored, clavate, thin-walled. Lamella edge sterile. Cheilocystidia 24–44 × 4–10 μm, cylindrical, hyaline, thin-walled. Pleurocystidia absent. Pileus covering a trichoderm; terminal cells 59–340 × 8–20 μm, erect, cylindrical to clavate, with rounded apices and slightly thick walls, containing light orange (6A3–A5) to greyish orange (6B3–B5) intracellular pigments. Stipe covering a cutis; terminal cells 55–150 × 8–16 μm, appressed, cylindrical to clavate, containing light orange (6A3–A5) to greyish orange (6B3–B5) intracellular pigments. Clamp connections present.

Habit and Habitat. Solitary to scattered on soil within the humus layer of broad-leaved forests dominated by *Fraxinus mandshurica*, *Quercus mongolica*, *Betula* spp., and *Ulmus* spp.

Known distribution: Jilin and Heilongjiang Provinces, China.

Additional specimens examined: CHINA. Jilin Province, Jiaohe City, Qianjin Forest Farm, 24 July 2022, X. Wang, FJAU78162, Ji’an City, Wunufeng National Forest Park, 8 August 2023, Q.R. Liu, FJAU78160; Changchun City, Jingyuetan National Forest Park, 28 July 2025, X.Y. Zhou, FJAU78172; 8 August 2025, W. Sun, FJAU78170; Huadian City, Hongshi National Forest Park, 30 July 2025, Y.F. Han, FJAU78173; Heilongjiang Province, Tahe County, Guqi Valley National Wetland Park, 25 July 2024, H. Cheng, FJAU78161.

Notes: Phylogenetically, *Lepiota sinocastanea* is positioned at the base of the *L. castanea* core clade and the *Lepiota* aff. *castanea* lineage, forming an independent sister group.

Although *L. sinocastanea* shares macroscopic features with other members of this complex, it can be distinguished by its relatively small basidiospores (7.8–9.8 × 2.7–3.8 μm, avl × avw = 8.6 × 3.3 μm, Q = 2.18–3.20). In contrast, both *L. castanea* [8.7–10.4(–11.1) × 3.0–3.7(–4.7) μm] and *L.* aff. *castanea* [(8.9–)9.4–11(–11.4) × 3.5–4.5 μm] observed in this study possess relatively large spores. Furthermore, the basidiospores of *L. sinocastanea* are non- to weakly dextrinoid, unlike the distinctly dextrinoid spores of *L. castanea* and *L.* aff. *castanea*. Macroscopically, *L. sinocastanea* is unique within this complex in possessing lamellae that discolor upon bruising. These morphological, chemical, and phylogenetic divergences strongly support its recognition as an independent species.

Additionally, our study revealed another phylogenetically distinct lineage within the *L. castanea* complex, designated here as *L.* aff. *castanea*. Since its basidiospore dimensions and other core morphological characteristics overlap substantially with those of *L. castanea*, and no stable diagnostic features have been identified, we provisionally adopt open nomenclature for this allied lineage pending further collections.

***Lepiota sirupa*** T. Bau & X.Y. Zhou, sp. nov.

MycoBank No: MB862977

**Figure 11:**
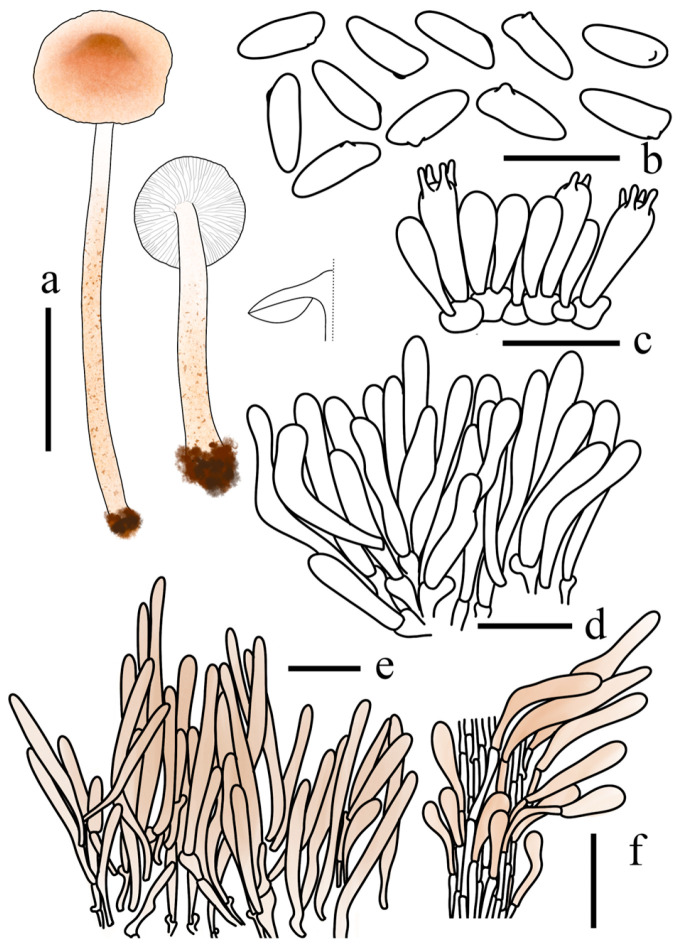
*Lepiota sirupa* (FJAU78261, holotype). (**a**) Basidiomata; (**b**) Basidiospores; (**c**) Basidia; (**d**) Cheilocystidia; (**e**) Pileus covering; (**f**) stipe covering. Scale bars: (**a**) =1 cm; (**b**) =10 µm; (**c**,**d**) =20 µm; (**e**,**f**) =50 µm.

Figure 9d–f and Figure 11

Holotype: CHINA. Jilin Province, Huadian City, Hongshi National Forest Park, 16 August 2024, X.Y. Zhou, FJAU78261.

Etymology: “*sirupa*” is derived from the Latin noun sirupus (syrup), referring to the brownish orange color of the pileus squamules, which resembles syrup.

Diagnosis: *Lepiota sirupa* is primarily characterized by its pileus densely clothed in floccose squamules with a color transition from light orange (6A2–A5) to brownish orange (7C6–C8); a stipe ornamented with squamules arranged in interrupted annular bands below the annular zone; non-dextrinoid, slender basidiospores (Qav = 2.67) appearing spurred or rectangular in profile; cylindrical to narrowly clavate cheilocystidia frequently possessing transverse septa; and a pileus covering structured as a trichoderm.

Basidiomata small. Pileus 0.9–1.1 cm in diam., convex to plano-convex, with an inconspicuous blunt umbo at the center; surface white, densely clothed in light orange (6A2–A5) to brownish orange (7C6–C8) floccose squamules. Context thin, white, unchanging when bruised. Lamellae free, crowded, interspersed with lamellulae, ventricose, white to cream; edge entire, concolorous. Stipe 2.7–2.9 × 0.1–0.3 cm, subcylindrical, slightly thickening towards the base, white to light orange (6A2–A5); surface nearly glabrous above the annular zone, lower portion densely ornamented with floccose to floccular squamules arranged in interrupted annular bands. Odour and taste not recorded.

Basidiospores (40/2/2) 6.9–8.2 × 2.5–3.4 μm, avl × avw = 7.5 × 2.8 μm, Q = 2.15–3.11, Qav = 2.67, spurred, rectangular to sub-triangular in profile, slightly ventricose on the adaxial side, long-ellipsoid in frontal view; wall hyaline, thin, non-dextrinoid. Basidia 16–28 × 5–9 μm, clavate, 4- (2-) spored, hyaline, thin-walled. Lamella edge sterile. Cheilocystidia 26–53 × 5–9 μm, cylindrical to narrowly clavate, occasionally with transverse septa, hyaline, thin-walled. Pleurocystidia absent. Pileus covering a trichoderm; terminal cells 76–219 × 6–13 μm, erect, cylindrical to clavate, with rounded apices and slightly thick walls, containing light orange (6A3–A5) to greyish orange (6B3–B5) intracellular pigments. Stipe covering a cutis; terminal cells 56–189 × 5–12 μm, appressed, cylindrical to clavate, containing light orange (6A3–A5) to greyish orange (6B3–B5) intracellular pigments. Clamp connections present.

Habit and Habitat. Solitary to scattered on soil within the humus layer of broad-leaved forests dominated by *Fraxinus mandshurica*, *Quercus mongolica*, *Juglans mandshurica*, *Betula* spp., and *Ulmus* spp.

Known distribution: Jilin Province, China.

Additional specimens examined: CHINA. Jilin Province, Huadian City, Hongshi National Forest Park, 16 August 2024, X.Y. Zhou, FJAU78262.

Notes: *Lepiota sirupa* is an orange-brown member of sect. *Stenosporae*. It shares macroscopic features and medium-sized basidiospores with *L. jilinensis* but differs in having cylindrical to narrowly clavate cheilocystidia (clavate to broadly clavate in *L. jilinensis*). Furthermore, the non-dextrinoid basidiospores separate *L. sirupa* from dextrinoid-spored relatives in this section, such as the *L. castanea* complex. Macroscopically, it is further characterized by unchanging white to cream lamellae and a stipe that does not discolor upon bruising.

**New record for China**

***Lepiota grangei*** (Eyre) Kühner, Bull. mens. Soc. linn. Soc. Bot. Lyon 3: 79 (1934)

**Figure 12:**
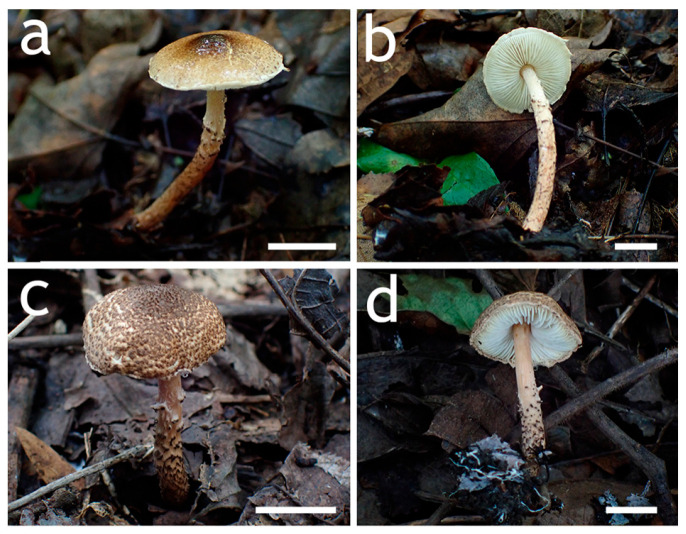
Basidiomata of *Lepiota grangei*. (**a**,**b**) FJAU78230; (**c**,**d**) FJAU78231. Scale bars = 1 cm.

**Figure 13:**
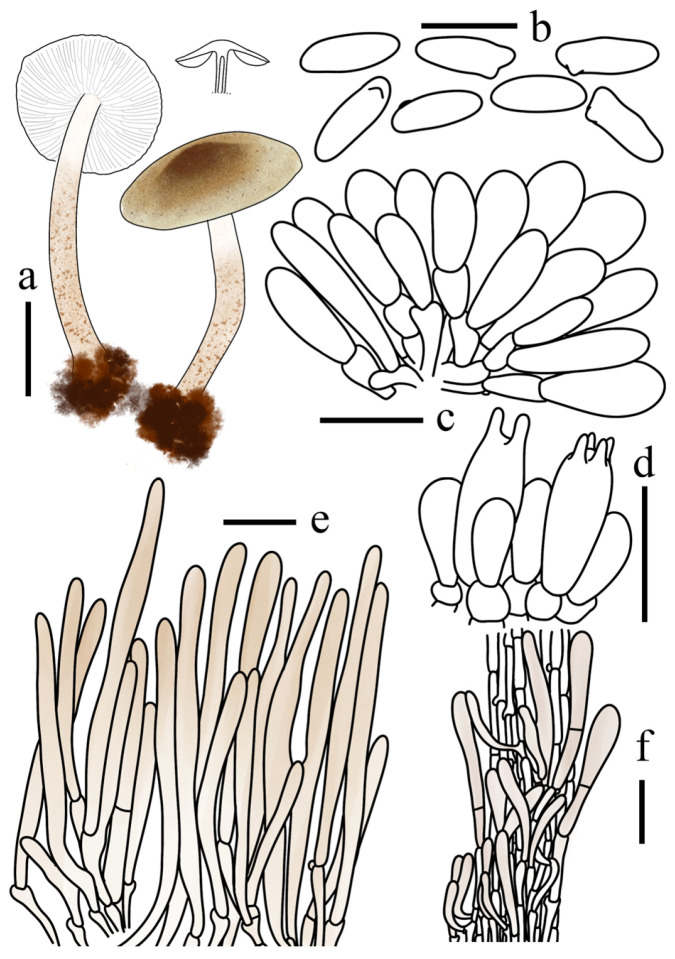
*Lepiota grangei* (FJAU78230). (**a**) Basidiomata; (**b**) Basidiospores; (**c**) Cheilocystidia; (**d**) Basidia; (**e**) Pileus covering; (**f**) stipe covering. Scale bars: (**a**) =1 cm; (**b**) =10 µm; (**c**,**d**) =20 µm; (**e**,**f**) =50 µm.

Figure 12 and Figure 13

Basidiomata small. Pileus 1.8–2.3 cm in diam., white to cream, covered with light olive brown (4D4–D8) squamules; scales at the center dense, tomentose, olive brown (4F7–F8). Context white. Lamellae free, crowded, interspersed with lamellulae of unequal length, ventricose, white to cream; edge entire, concolorous. Stipe 3.5–5.2 × 0.3–0.4 cm, subcylindrical, cream; surface nearly glabrous above the annular zone, lower portion densely covered with olive brown (4F7–F8) granular squamules below, arranged in intermittent bands. Odour and taste not recorded.

Basidiospores (40/2/2) 9.6–11.4 × 3.3–3.7 μm, avl × avw = 10.3 × 3.5 μm, Q = 2.62–3.29, Qav = 2.89, subtriangular in profile with a distinct suprahilar depression and a prominent basal spur, subfusiform in frontal view; ventral side slightly ventricose; dorsal side ventricose but often depressed in the lower-middle part; apex gradually tapering; wall hyaline, slightly thick, dextrinoid. Basidia 20–30 × 6–11 μm, clavate, 4- (rarely 2-) spored, hyaline, thin-walled. Lamella edge sterile. Cheilocystidia 26–38 × 6–8 μm, clavate, often with a simple septum, hyaline, thin-walled. Pleurocystidia absent. Pileus covering a trichoderm; terminal cells 116–330 × 10–17 μm, erect, cylindrical to clavate, with up to 3 clampless septa, with rounded apices, slightly thick walls, containing greyish yellow (4B2–B3) to light blond (4C2–C3) parietal and intracellular pigments. Stipe covering a cutis; terminal cells 52–193 × 7–15 μm, appressed, cylindrical to clavate, containing light blond (4C2–C3) intracellular pigments. Clamp connections present.

Known distribution: Widespread in Europe (e.g., Germany and the Netherlands), also recorded from North America (USA) and South America [12,22]. In Asia, it has been documented in Thailand, South Korea [62], and Northern China (Jilin Province, this study).

Specimens measured: CHINA. Jilin Province, Huadian City, Hongshi National Forest Park, 16 August 2024, T.Y. Zhang, FAJU78230; Jilin City, Songhua Lake Scenic Area, 20 September 2024, J.L. Wei, FAJU78231.

Notes: *Lepiota grangei* is a well-documented species in Europe, typically characterized by pileus squamules with more pronounced green, bluish-green, or grayish-blue tones [12,63]. In contrast, our specimens from Northern China exhibit a light olive brown to olive brown color range. This variation likely reflects intraspecific plasticity, as the pileus color of this species has previously been described as varying from blue-green to brownish-green [63].

Phylogenetically, members of subclade II share these distinctive greenish-brown pileus tones. Geographically, *L. griseovirens* is known from Africa and Europe [11,63], *L. poliochloodes* from Europe and Thailand [7,63], and *L. brunneoolivacea* from Benin [11]. In China, only three species—*L. grangei*, *L. hongshiensis*, and *L. pilodes*—possess greenish-brown pileus squamules. However, *L. hongshiensis* is easily distinguished by its exceptionally small basidiospores (5.9–7.1 × 2.9–3.5 μm) and the absence of cheilocystidia. Although *L. pilodes* also produces relatively small spores (7.5–8.9 × 2.8–3.4 μm), it lacks septa in the terminal cells of the pileus covering. *L. grangei* is recognized by its relatively large basidiospores (9.6–11.4 × 3.3–3.7 μm), clavate cheilocystidia, and the presence of conspicuous septa in the terminal cells of the pileus covering.

**Key to the 17 species of** ***Lepiota*** **sect.** ***Stenosporae***** characterized by yellowish-orange to orange-brown pileus squamules**1. Pileus covering a cutis*L. boudieri*1’. Pileus covering a trichoderm22. Lamellae pale yellow to greyish yellow*3*2’. Lamellae white to cream*4*3. Basidiomata turning dark brown to black upon drying*L. flavonigrescens*3’. Basidiomata unchanging upon drying*L. citrophylla*4. Basidiospores inamyloid, weakly dextrinoid, or reaction unknown54’. Basidiospores distinctly dextrinoid125. Basidiospores with average length > 9.0 μm65’. Basidiospores with average length < 9.0 μm76. Basidiospores narrowly cylindrical, 11.5–14.1 × 3.3–4.7 μm (avl × avw = 12.9 × 3.9 μm), Q = 2.92–3.93, Qav = 3.31*L. dolichospora*6’. Basidiospores broader, 8.5–11.0 × 3.5–4.5 μm (avl × avw = 9.7 × 4.0 μm), Q = 2.00–2.86, Qav = 2.48*L. subcastanea*7. Basidiospores with average length < 7.0 μm87’. Basidiospores with average length > 7.0 μm108. Distributed in South Asia (Pakistan)*L. brunneoaurantia*8’. Distributed in East Asia (China)99. Basidiospores relatively small (avl × avw = 5.7 × 2.8 μm); cheilocystidia narrowly clavate to clavate*L. microstenospora*9’. Basidiospores relatively large (avl × avw = 6.5 × 3.2 μm); cheilocystidia narrowly clavate, rarely narrowly utriform or fusiform*L. mandarina*10. Lamellae changing colour upon bruising*L. sinocastanea*10’. Lamellae unchanging upon bruising1111. Cheilocystidia 19–35 × 9–18 μm, clavate to broadly clavate *L. jilinensis*11’. Cheilocystidia 26–53 × 5–9 μm, cylindrical to narrowly clavate*L. sirupa*12. Basidiospores 7.5–14.0 μm in length1312’. Basidiospores 4.7–7.5 μm in length1413. Cheilocystidia narrowly clavate to fusiform or utriform*L. castanea*13’. Cheilocystidia cylindrical to narrowly clavate *L. alopochroa*14. Distributed in Asia (Pakistan)*L. brunneopileata*14’. Distributed in Africa (Benin)  1515. Basidia with distinctly long sterigmata, 5–10(–20) μm*L. longisterigmata*15’. Basidia without distinctly long sterigmata1616. Stipe apex white to pale yellow*L. flavostipitata*16’. Stipe apex white to cream*L. aurantiicolor*

Note: *Lepiota ignicolor* Bres. is excluded from this key due to unresolved taxonomic uncertainties. Morphologically, its basidiospore size range and cheilocystidia shape significantly overlap with those of *L. castanea*. Regarding molecular data, the sequence labeled as L. ignicolor in our phylogenetic tree (GenBank accession: AY176472) [4] lacks a corresponding morphological description for its voucher specimen. Conversely, available morphological descriptions of this species [19] lack supporting molecular data. Given this discrepancy, the true taxonomic concept of *L. ignicolor* remains ambiguous. The morphological data utilized in this key are derived from the present study and the following cited literature [7,11,18,19,35].

## 4. Discussion

### 4.1. Phylogenetic Framework and Sectional Delimitation

Our phylogenetic analysis, based on a combined four-gene dataset (ITS, nrLSU, *rpb2*, and *tef1-α*), demonstrates that *Lepiota* sect. *Stenosporae* constitutes a well-supported monophyletic group within *Lepiota* (97/1). The section is clearly bifurcated into two major evolutionary lineages based on pileus covering micromorphology: Clade A (trichoderm) and Clade B (cutis-like) [43,64]. This topology is highly consistent with previous taxonomic frameworks [7,9,10,11], further validating the phylogenetic significance of the pileus covering in sectional delimitation.

Within Clade A, Subclade I exhibits high genetic divergence despite pronounced macroscopic similarities. Except for a few color-specialized taxa like the white-toned *L. subalba* [48] and purple-toned *L. tyrianthina* [11], members of this subclade possess similar yellowish-orange to orange-brown pileus scales. These macroscopic similarities among genetically distinct species present a challenge for field identification. Consequently, the taxonomic key provided herein is intended to assist in species-level differentiation.

### 4.2. Evolutionary Complexity and Phenotypic Diversity

In contrast to the well-resolved Subclade I, the remaining lineages in Clade A exhibit more complex evolutionary patterns. Subclade II is generally characterized by greenish-brown tones. Meanwhile, Subclade III, comprising three known species, further exemplifies the discordance between macromorphology and phylogeny. Although *L. citrophylla* [48] and *L. flavonigrescens* [11] share yellowish-orange to orange-brown pilei with members of Subclade I, they are readily separated by their distinct yellow-toned lamellae—a key diagnostic feature absent in the latter. In contrast, *L. pilodes* exhibits greenish-brown tones typically associated with Subclade II; however, our topology reveals that it does not cluster with the Subclade II lineage, instead phylogenetically aligning with the yellow-gilled species of Subclade III. This suggests that comparable color phenotypes (such as greenish-brown or orange-brown) have likely evolved independently across different lineages within the section.

Furthermore, Subclade IV (*L. tomentella*) is distinguished by grey-brown tones with a pinkish tinge, yet it exhibits significant intraspecific genetic variation, indicating potential cryptic diversity. In contrast, Clade B displays a much broader color spectrum, encompassing dark brownish-yellow, orange-brown, and pinkish-grey tones. This distribution of color phenotypes across the section underscores the taxonomic complexity within sect. *Stenosporae*. Further studies incorporating genomic-scale data are required to precisely resolve the evolutionary trajectories of these complex lineages.

### 4.3. Taxonomic Considerations of the Lepiota castanea Complex

Historically, the *Lepiota castanea* complex has received extensive attention due to the high variability in basidiospore dimensions and other microscopic features. The original description recorded relatively small basidiospores (8–9 µm) [65], whereas it was subsequently proposed, based on morphological boundaries, that the large-spored lineage (13.5–14.5 µm) be delimited as a distinct species, *L. rufidula* Bres. [66]. Conversely, based on extensive morphological studies of European material, a continuous distribution of spore sizes (7.0–14.0 µm) was observed, leading to the adoption of a broad species concept [12].

Our molecular phylogenetic data provide a clear genetic basis for understanding this variation. The analysis reveals that the complex is composed of at least three genetically independent evolutionary lineages, indicating that the morphological variations observed in previous studies correspond to distinct evolutionary branches.

The spores of the new species *L*. *sinocastanea,* average 8.6 × 3.3 µm, aligning closely with the original records of *L. castanea* [65]. Its basal evolutionary position within the complex, its non- to weakly dextrinoid reaction in Melzer’s reagent, and the distinct discoloration of the lamellae upon bruising strongly support its recognition as an independent East Asian lineage. Furthermore, another Chinese lineage (referred to herein as *L*. aff. *castanea*) possesses relatively large spores (averaging 10.0 × 3.9 µm), which is consistent with the morphological concept of the large-spored taxa. Given the biogeographic isolation between East Asia and Europe, and the current lack of sequences from the type locality of *L*. *rufidula*, we conservatively adopt open nomenclature for this lineage, pending further validation through broader global sampling.

### 4.4. Conclusions

Based on field surveys in Northeast China, this study describes six new species and one new regional record within *Lepiota* sect. *Stenosporae*. These findings expand the known species diversity of this section in East Asia and demonstrate that macroscopically similar lineages actually represent distinct evolutionary branches. Because macroscopic traits often exhibit convergent evolution, resolving complex groups in this section requires integrating multi-locus phylogeny with stable micro-morphological features (e.g., basidiospore dimensions and cheilocystidia shapes). While this study clarifies the taxonomy of several East Asian taxa, further global sampling remains necessary to fully resolve the evolutionary boundaries within sect. *Stenosporae*.

## Data Availability

All the sequences have been deposited in GenBank (https://www.ncbi.nlm.nih.gov, accessed on 24 March 2026) and MycoBank (https://www.mycobank.org, accessed on 24 March 2026). For peer-review purposes, the concatenated phylogenetic alignment and detailed specimen metadata are temporarily available in the Zenodo repository (DOI https://doi.org/10.5281/zenodo.19253651, accessed on 8 May 2026).
